# Patterns of Non-adherence to Supplementation with Calcium and Vitamin D in Persistent Postmenopausal Women Are Similar at Start and 1 Year Later: A Qualitative Longitudinal Study

**DOI:** 10.3389/fphar.2016.00339

**Published:** 2016-09-30

**Authors:** Tereza Touskova, Magda Vytrisalova, Vladimir Palicka, Tereza Hendrychova, Yang-Ti Chen, Leos Fuksa

**Affiliations:** ^1^Department of Social and Clinical Pharmacy, Faculty of Pharmacy in Hradec Kralove, Charles University in PragueHradec Kralove, Czech Republic; ^2^Faculty of Medicine, OsteoCentre, Institute of Clinical Biochemistry and Diagnostics, Charles University in Prague, University Hospital in Hradec KraloveHradec Kralove, Czech Republic

**Keywords:** medication adherence, patient adherence, longitudinal studies, medication event monitoring system (MEMS), drug holidays, osteoporosis, calcium supplementation, electronic monitoring

## Abstract

**Background:** Osteoporosis is a chronic disease and adherence can fluctuate over time. Therefore, longer observation is necessary to investigate the stability of patients' adherence. The study aim was to compare the overall adherence (OA) to supplementation with the fixed combination of calcium and vitamin D (Ca/D) in postmenopausal women at baseline and after 1 year, and to evaluate the fluctuation of the OA in individual months. Furthermore, we studied whether adherence is influenced by signing of informed consent and routine medical check-up.

**Methods:** This was a longitudinal, observational study. The data were obtained from the Osteocenter of University Hospital in Hradec Kralove, Czech Republic. Adherence was measured using electronic bottles type Medication Events Monitoring System (MEMS). The study was carried out in two 3-month periods; the baseline in 2013 (signing of informed consent while medical check-up) and the follow-up (medical check-up) in 2014. The adherence and adherence-related outcomes were studied in patients who had initiated osteoporosis treatment and were persistent.

**Results:** 21 (49%) out of 43 patients who avoided drug dispenser and were persistent both at baseline and at follow-up, completed the study and were included. Median age was 76. Evaluating the whole 3-month periods, the OA did not differ significantly at baseline and at follow-up, the OA was 71 and 68%, respectively. However, the adherence in month 1 at baseline was significantly higher than the adherence in month 2 at baseline (*p* < 0.001) and also than the adherence in month 1 at follow-up (*p* = 0.010). Analysing the study period without month 1, a stable adherence was observed in 48% of patients. About 33% of doses were omitted at baseline and 34% at follow-up. As many as 71% of the patients took drug holidays at baseline, and 76% at follow-up.

**Conclusion:** The OA was insufficient, around 70% both at baseline and at follow-up. One half of the patients showed a stable adherence. The patterns of non-adherence were very similar at follow-up. Signing of the informed consent seems to act as bias more than regular medical check-up.

## Introduction

Osteoporosis is defined as a chronic systemic disease of skeleton characterized by low bone mineral density which resulted in an increased risk of fractures. Changes in microarchitecture of bone tissue can also significantly contribute to bone fragility (Kanis et al., 2013).

A lot of medications for the treatment of osteoporosis have been proven to reduce fracture risk in postmenopausal women. Antiresorptive drugs, such as bisphosphonates, which prevent accelerated decrease of bone mass associated with age, are widely recommended and used (Kanis et al., 2013). All treatment regimens should include adequate intakes of calcium and vitamin D (Ca/D). Daily doses of 1000 mg of calcium and 800 international units (IU) of vitamin D or even higher can be generally recommended (Kanis et al., 2013). According to the study of Tang et al. (2007), supplementation with Ca/D was associated with a 24% reduction in the fracture risk in patients sufficiently adherent to the supplements.

Non-adherence to medical therapy is a widespread public health problem. It is estimated that only a half of the patients comply with a long-term therapy (Kanis et al., 2013). Especially in chronic asymptomatic diseases, overcoming of non-adherence presents a particular challenge (Kanis et al., 2013). This fact is well-documented by the results of recent studies concerning the treatment of osteoporosis, in which the adherence to long-term Ca/D supplementation ranges between 30 and 75% (Sanfelix-Genovés et al., 2009; Castelo-Branco et al., 2010; Díez et al., 2012; Touskova et al., 2015).

The work of Voils et al. (2011) which deals with the methodology for measuring adherence concluded that longitudinal evidence is needed to illuminate whether episodic or chronic non-adherence is more common. As it is possible that patients who are non-adherent in cross-sectional studies will become adherent year after year and vice versa (Ngui et al., 2013), and that adherence may vary over the time, repeated measures are necessary to identify non-adherent patients more adequately (Tesoriero et al., 2003).

In the field of adherence evaluation, one of the most important problems is that traditional methods (pill counts, questionnaires, patients' diaries) have repeatedly been shown to overestimate adherence (Shi et al., 2010a,b). Further, adherence could be unwittingly overestimated when measured retrospectively (Zeller et al., 2008a).

A reliable evaluation of poor adherence, including early discontinuation, can be performed over a long term follow-up from the timing of refilled prescriptions in population databases. However, refills do not capture medication-taking habits, including the exact time when the patient discontinued taking the medication. Thus, the most reliable method for measuring the adherence is the use of Medication Event Monitoring System (MEMS; Osterberg and Blasche, 2005). Although this objective method is also connected with some questionable aspects, these could be mostly overcome by means of appropriate design of the study.

There could be an influence of signing a written consent as patients who provide it might be more motivated and adherent than those who refused to participate (Cheng et al., 2004). Some works show a mild improvement of adherence in connection with a medical check-up (Feldman et al., 2007; Gillespie et al., 2014). Nevertheless, it is necessary to further investigate these hypotheses.

Based on abovementioned facts our study aimed (Kanis et al., 2013) to compare the adherence to Ca/D at baseline and after 12 months of the treatment in postmenopausal women with osteoporosis (Tang et al., 2007). To evaluate the fluctuation of the adherence in individual months and (Touskova et al., 2015) to find out if the adherence is influenced by the entry to the study (written consent) and medical check-up.

## Methods

### Study design, setting, and participants

A longitudinal, observational study was performed in secondary care female patients at the risk of osteoporosis-related fracture. The study was carried out in two periods lasting 3 months each; the baseline from June to September 2013 and the follow-up from June to October 2014. Study participants were recruited from the outpatient Osteocenter in University Hospital in Hradec Kralove, Czech Republic which specializes in providing care to patients with osteoporosis.

Detailed methodology including the inclusion and exclusion criteria are described elsewhere (Touskova et al., 2015). Briefly summarized, the adherence to a fixed combination of calcium and vitamin D (Ca/D), preparation Caltrate 600 mg/400 IU D3® (tablets) was evaluated. One tablet once a day was prescribed to all participants. Postmenopausal women older than 55 years with the diagnosis of osteoporosis and concurrent treatment with oral ibandronate were recruited. Exclusion criteria were non-initiation and non-persistence with the Ca/D treatment, failure to adhere to the study protocol and the use of a drug dispenser. Each patient got a MEMS bottle containing 90 tablets of Ca/D (amount for 3 months) in both rounds. The monitoring of the adherence was performed in the same way at baseline and at follow-up.

Electronic monitoring using MEMS is an objective indirect method of measuring adherence, indicating when a medication has been used. Each opening of the bottle is detected and obtained records are analyzed. Although a patient may just open the bottle without use of the medication or may use other than the prescribed dose, distortion of results by these limitations can be estimated (e.g., multiple openings in a short time can indicate experimenting with the MEMS-bottle, openings once a week can indicate use of weekly drug dispenser).

**Figure 1:**
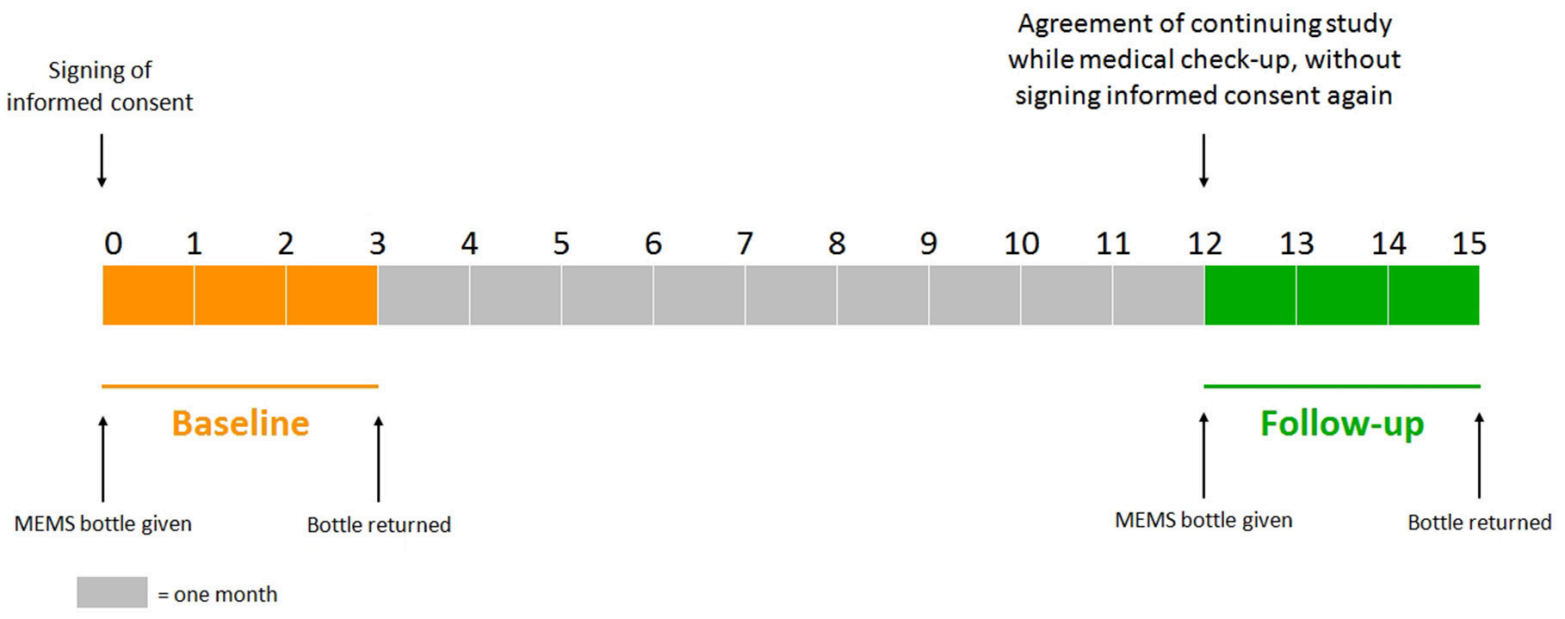
**Study design**.

The study protocol has been approved by Ethics Committee, University Hospital Hradec Kralove. Each patient agreed with her inclusion in the study by signing informed consent during a regular medical check-up in the osteocenter at baseline. After 3 months of monitoring, the patient gave the MEMS bottle back (without medical check-up). If the patient had agreed with her continuation in the study, she would be provided with the MEMS bottle again during a regular medical check-up 12 (±1) months after the beginning of the baseline without signing informed consent again (at the moment). The MEMS bottle was returned to the osteocenter after 3 months of the follow-up monitoring (without medical check-up), see Figure 1.

### Outcome measures

In this article, the term adherence is used. It is a synonym for compliance (closeness to treatment recommendation, often simplified as the number of doses indeed taken related to the number of prescribed doses), however the term compliance embeds a more paternalistic doctor/patient relationship. The term persistence expresses duration of the treatment, i.e., how long the medication is taken (Dezii, 2001; Vrijens et al., 2012; Kanis et al., 2013). We studied adherence-related outcomes in patients who had initiated osteoporosis treatment and were persistent both at baseline and at follow-up i.e., implementation phase (Vrijens et al., 2012). Adherence ≥80% was considered as good, adherence <80% as poor (Cramer et al., 2007).

Definitions:

- **Overall adherence (OA, primary outcome measure):** ratio of the number of container openings to the number of prescribed doses. The OA can exceed 100%.
- **Difference in overall adherence:** The overall adherence at follow-up period minus the overall adherence at baseline period. The change of 20% or more was considered as a marked increase or marked decrease.
- **Stable adherence:** OA in each study month did not exceed 10% difference from the mean OA of the whole study period.
- **Single or sequentially missed multiple doses (omissions of dose):** We focused on the omissions of a dose on a single day, two consecutive days and drug holidays, defined as a sequence of at least three consecutive days without taking the drug. We defined the omission of a dose on a single day (24 h) as Ca/D-free interval from 3 a.m. to 3 a.m. (the next day). A similar rule was used to define the omission of more than 1 day (Vrijens et al., 2012).
- **Non-persistence:** gap in the use of Ca/D to be 30 days or more (Cramer et al., 2007; Kothawala et al., 2007).

### Data processing and statistical analysis

Most variables did not follow a normal distribution and therefore non-parametric statistics were applied. Changes in adherence-related outcomes in two time periods, i.e., follow-up vs. baseline, pairs of individual months, were tested by using Related-samples Wilcoxon Signed Rank test. Statistical analyses were calculated using PASW software (version 18.0). *P* < 0.05 was considered statistically significant.

## Results

**Figure 2:**
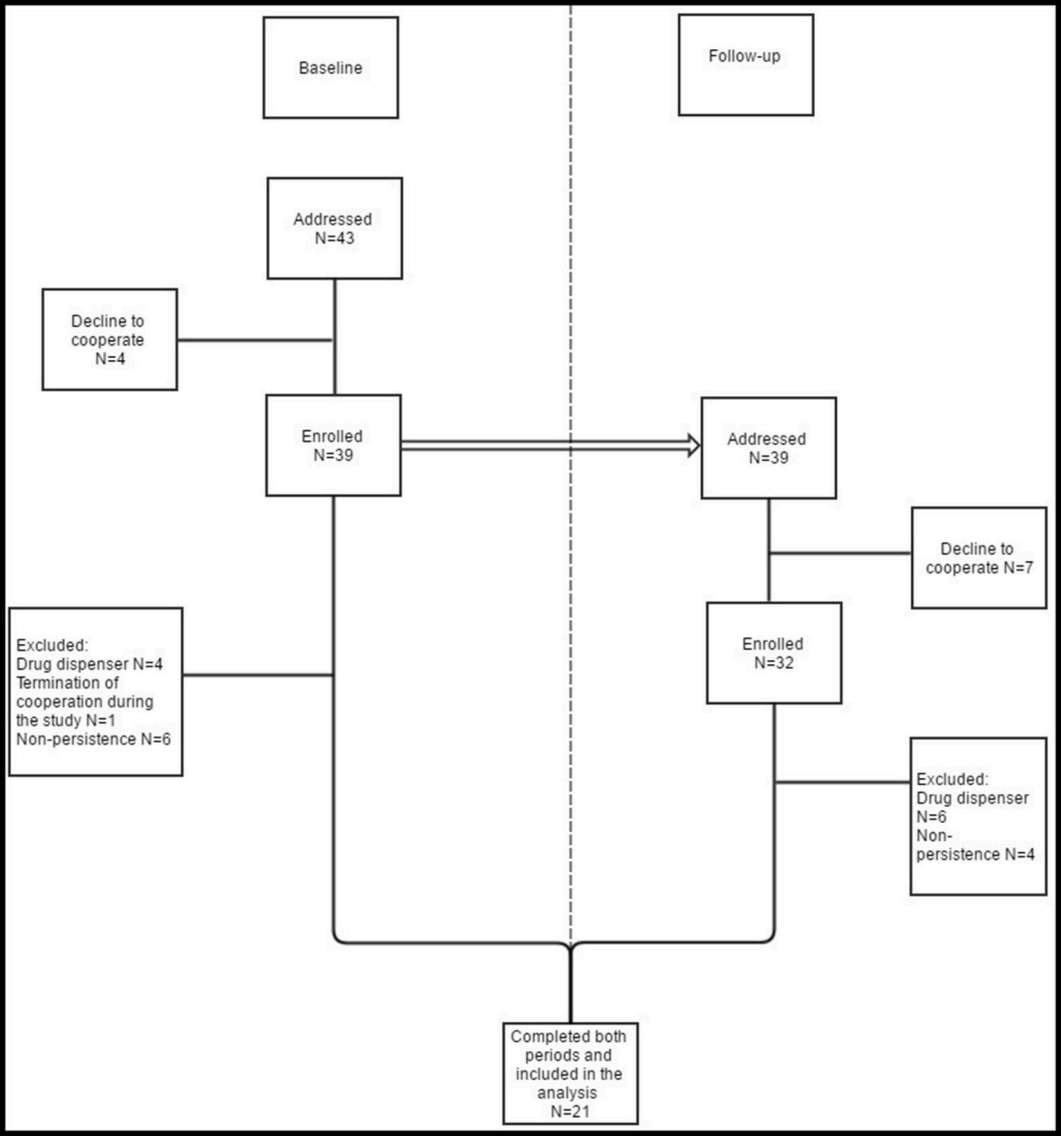
**Study organization**. Patients who met the exclusion criteria at baseline and/or at follow-up were not included in the analysis. The same patients who were enrolled and monitored at baseline (even those finally excluded) were addressed at follow-up. Therefore, patients who were excluded at baseline, were enrolled at follow-up. Although they did not meet the exclusion criteria at follow-up, they cannot be (due to non- persistence and/or use of drug dispenser at baseline) included in the analysis.

Of 43 (39) patients who were addressed (enrolled and monitored) at baseline, as many as 21 (49%) completed both periods and met the inclusion criteria. These 21 patients did not use drug dispenser and were persistent both at baseline and at follow-up and therefore were included in the present analysis. The flow of patients through the study and the reasons for exclusion from the analysis are shown in Figure 2. Median age (minimum-maximum) of the included patients was 76 (60–83) years. The number of patients excluded by reason of non-persistence was 6/4 (baseline/follow-up). From this count only one patient was non-persistent in both rounds. A few patients (4/7) refused to participate in the study. In the follow-up they stated reasons such as a planned holiday or inconvenience with MEMS bottle. From seven patients who refused to cooperate in the follow-up, only one was non-persistent at baseline. As many as 4/6 patients were excluded by reason of using a drug dispenser (4 patients from the baseline continued with this habit and 2 others started with it). None of these patients was non-persistent.

No multiple openings of the MEMS container within a period of ≤15 min were observed.

### Difference in overall adherence

**Figure 3:**
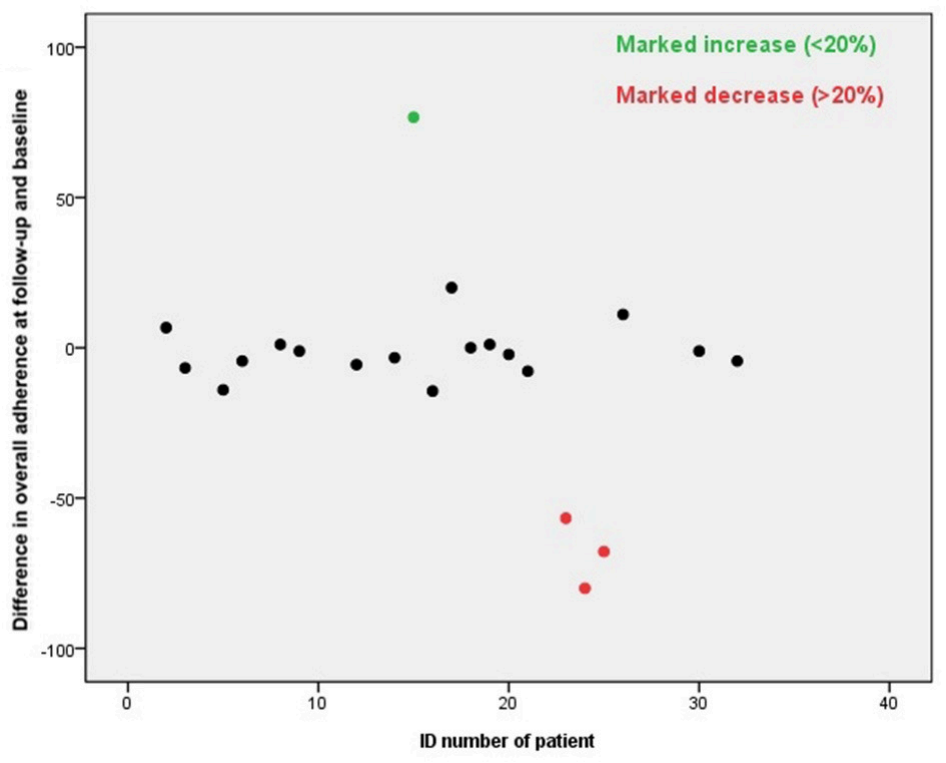
**Difference in mean overall adherence at follow-up and baseline**.

The overall adherence did not differ significantly at baseline period and the follow-up, the overall adherence was 71 and 68%, respectively. The adherence of 1 patient (5%) increased markedly and of 3 patients (14%) decreased markedly, see Figure 3. A good adherence (≥80%) was observed in 62% of the patients at baseline and 57% at follow-up. The overall adherence at follow-up was 78% in patients showing good adherence at baseline, compared to 51% in patients with a poor adherence at baseline.

### Stability in adherence by study month

**Figure 4:**
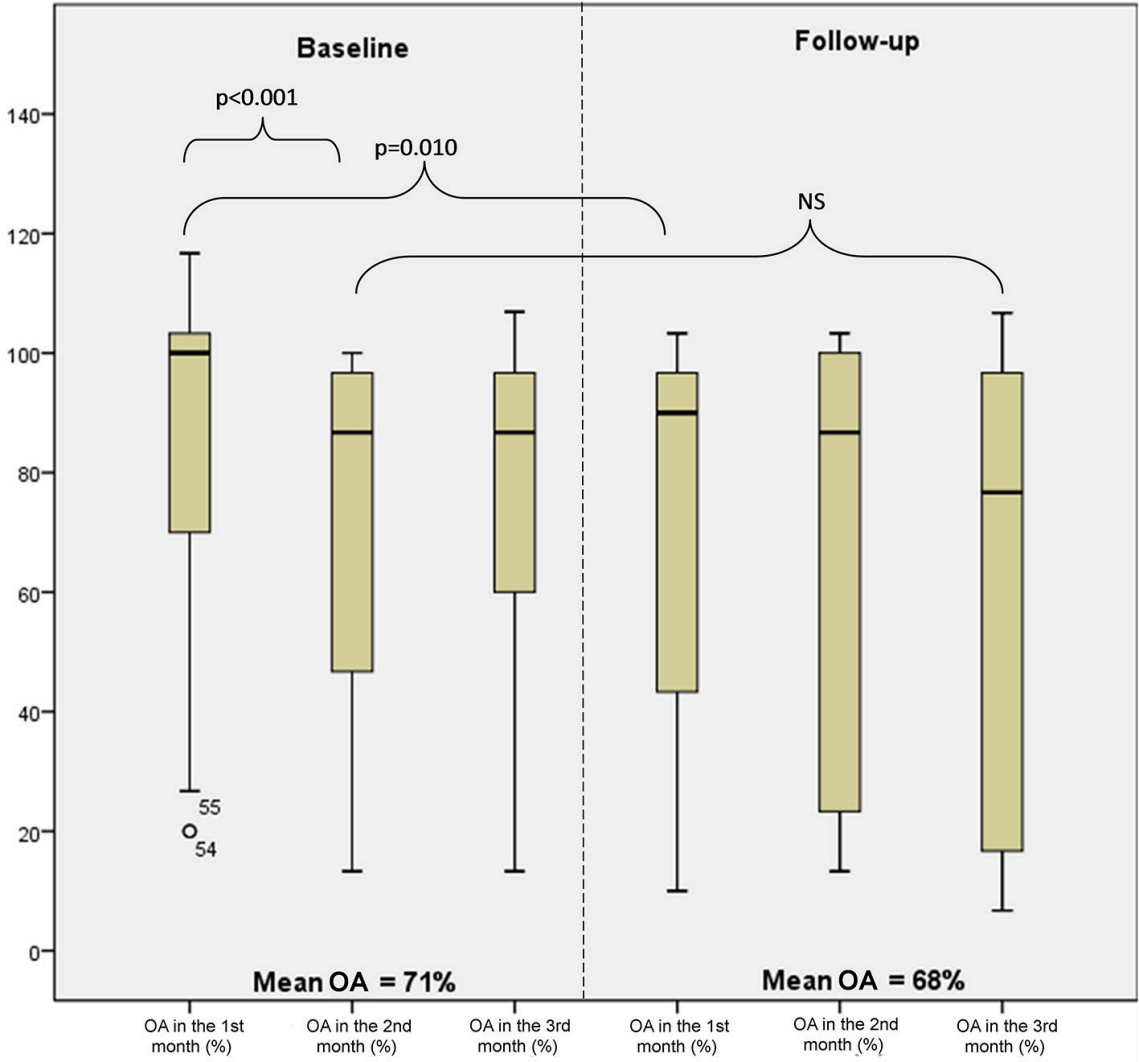
**Levels of overall adherence (OA) at baseline and follow-up by month (minimum, 25th percentile, median, 75th percentile, and maximum)**.

Changes in the overall adherence by a study month are shown in Figure 4. The adherence in month 1 at baseline was significantly higher than the adherence in month 2 (*p* < 0.001) and also significantly higher than the adherence in month 1 at follow-up (*p* = 0.010). On the contrary to month 2 vs. month 1 at baseline, the differences in the adherence when evaluating the pairs of consecutive months were not significant.

As many as 33% of patients maintained a stable adherence. When analyzed the study period without month 1, i.e., from month 2 at baseline to month 3 at follow-up, a stable adherence was observed in 48% of patients.

### Non-adherence patterns

**Figure 5:**
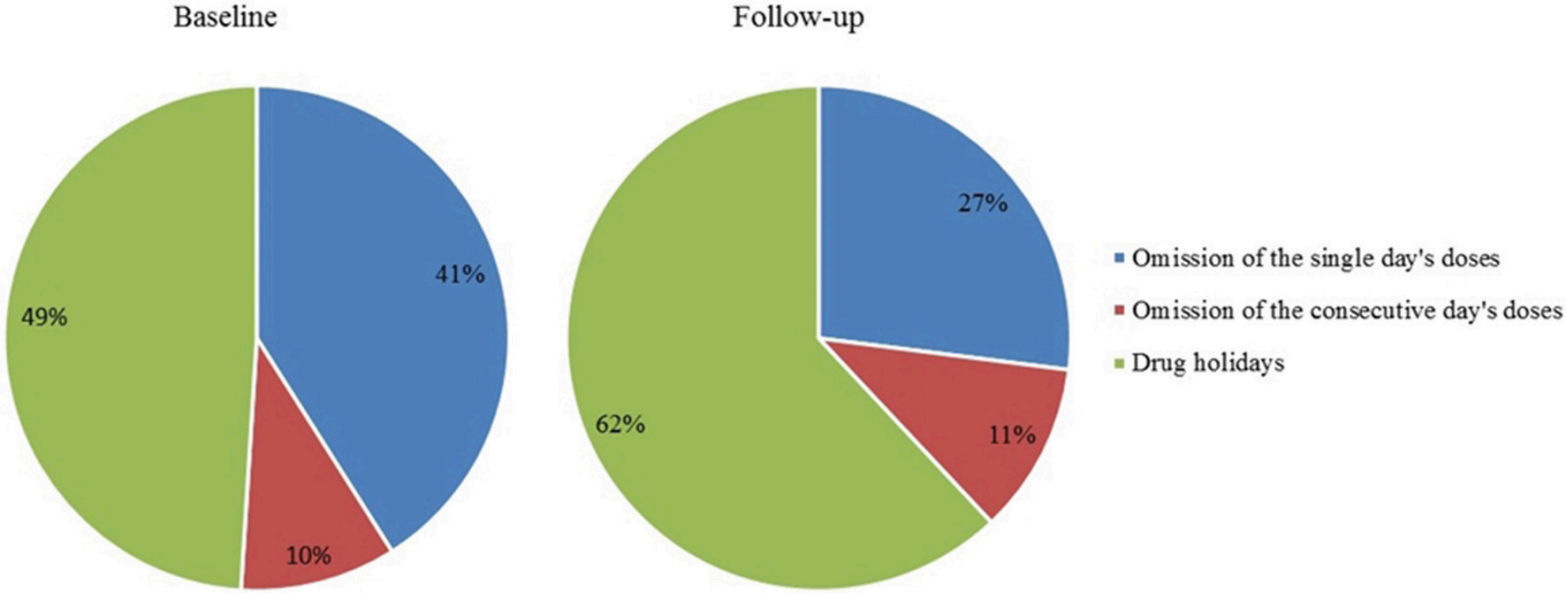
**Omitted doses within single-day omission, 2-day omission, and drug holidays (%); all omitted doses represent 100%**. Drug holidays, a sequence of at least 3 consecutive days without taking the drug.

As many as 33% of doses were omitted at baseline and 34% at follow-up. The categorizations of these omissions at baseline and follow-up are showed in Figure 5.

As many as 71% of the patients took drug holidays at baseline and 76% at follow-up. Patients without drug holidays were fully compliant, the overall adherence was 102% at baseline and 98% at follow-up.

**Figure 6:**
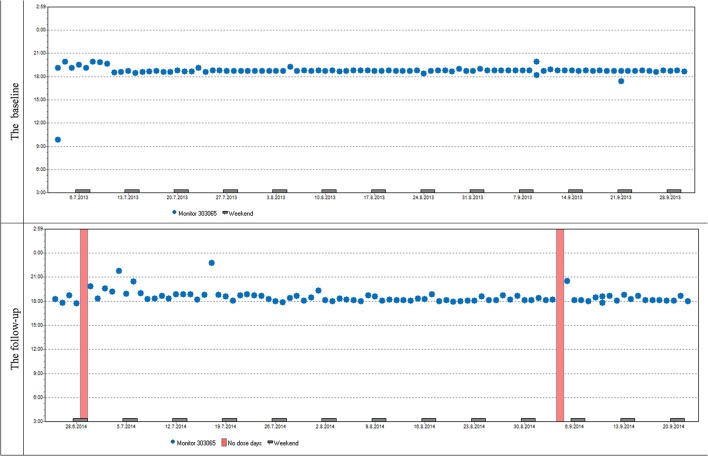
**Case report 1:** Overall adherence of the patient at baseline and follow-up is very high, adherence over time is stable at baseline and follow-up. The patient used her medication regularly in the evening as prescribed by her doctor. The same trend of use was found in most participants both in timing and dosage.

**Figure 7:**
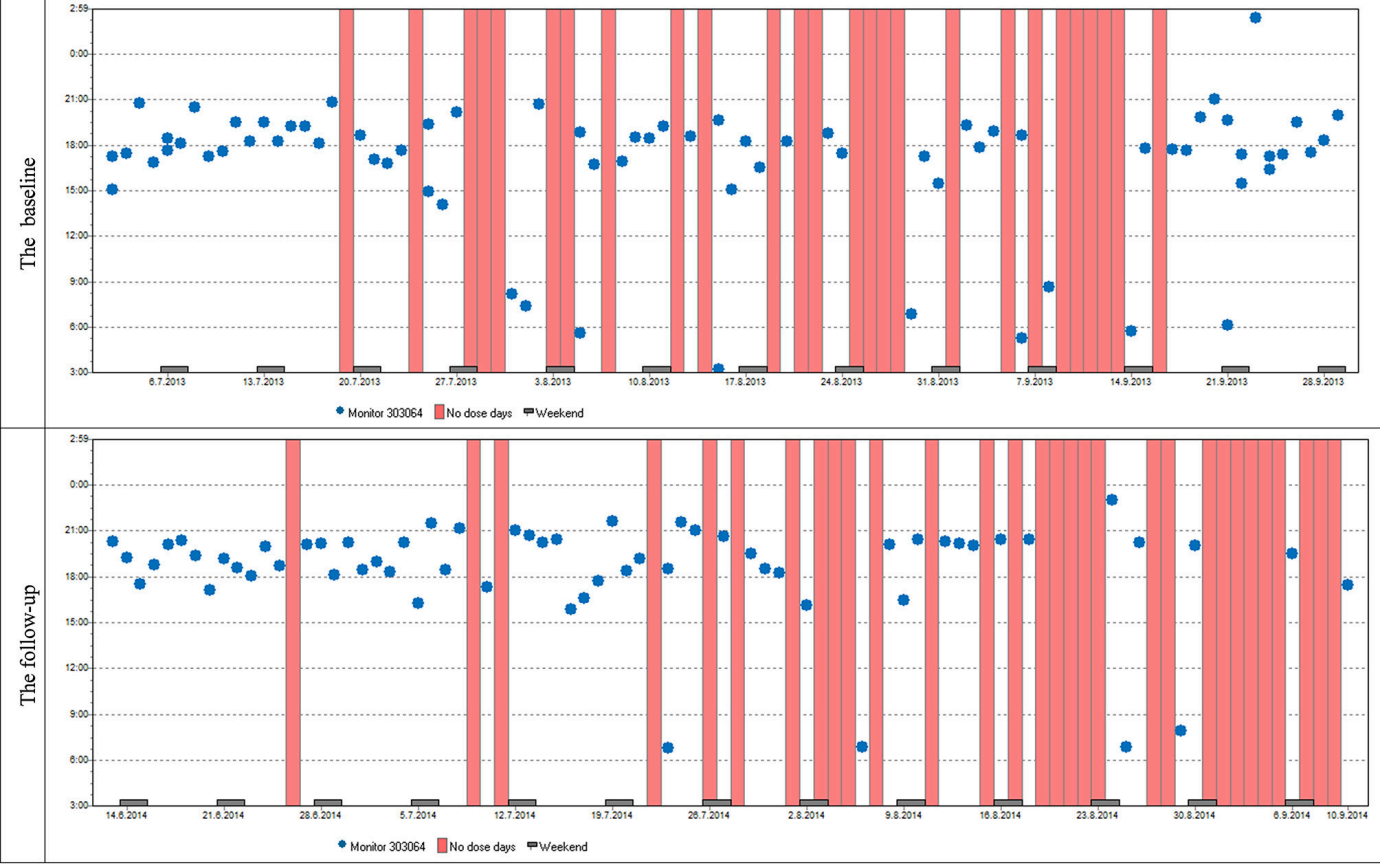
**Case report 2:** Just like the previous patient, she used her medication mostly in the evenings. Although her overall adherence is lower, it is still stable with the difference in the overall adherence being insignificant. The trend of omitted doses resulting in drug holidays is similar as well; we can see dose omissions on single days, two consecutive days, and eventually drug holidays, whose frequency is slightly increased at follow-up.

Variables describing patterns of non-adherence such as single-day omissions, 2-day omissions, drug holidays, and the longest drug holidays did not differ significantly at baseline and at follow-up. To demonstrate similar patterns in adherence at baseline and follow-up in most patients we present two cases (Figures 6, 7).

## Discussion

The presented longitudinal prospective study was conducted to investigate the adherence over the period of more than 1 year. Because adherence can fluctuate over time, we observed patients non-persistent at baseline also during the follow-up, although it is unusual (especially in database studies; Kothawala et al., 2007; Lekkerkerker et al., 2007). The majority of patients non-persistent at baseline was persistent at follow-up, which generates the signal that in many cases patients restart successfully with the treatment. Non-persistence, up to a point, can be thus considered as extremely long drug holidays.

The design of the study has been approached unusually in comparison with other studies (Cheng et al., 2004; Parker et al., 2007)—the monitoring was not held during the whole year but with a nine-month pause. The reason for this was our attempt to compare the influence of the entry to the study (informed consent signing) and the medical check-up (the first and the twelfth month of the study) on adherence of patients. Some authors state a mild improvement of adherence in connection with the medical check-up (Feldman et al., 2007; Gillespie et al., 2014), but this behavior was observed only partly in our study. Patients were significantly more compliant the first month after the medical check-up compared to following 2 months at baseline. On the other hand, adherence after the medical check-up did not change in comparison with next 2 months at follow-up. Thus, the significantly higher adherence in the first month after study entry is more likely to be linked with the signing of informed consent than the medical check-up. It seems that even though patients have not known about electronic monitoring of adherence, the very fact of signing the informed consent could play the role of bias. Based on these findings we suggest that further studies, similar to ours, should analyse adherence after the initial period, as it is probably influenced by bias of signing the informed consent.

The overall adherence (OA) was insufficient, around 70% both at baseline and follow-up. It changed markedly only in one fifth of the patients at follow-up. If we do not consider adherence in the first month, which suggests the influence of social desirability bias, we observed a stable adherence in one half of the patients during particular months (max ± 10% of the mean counted for all 5 months); one half of the patients showed higher fluctuation.

As an adequate intake of calcium and vitamin D is essential for the successful treatment of osteoporosis and our results confirm an insufficient adherence to the supplementation of Ca/D, we emphasize the role of diligent patient education in the field of an appropriate diet, rich in these key nutrients, in clinical practice.

We observed good adherence (≥80%) in fewer patients (around 60%) than Cheng et al. (around 80%), who studied adherence to statin therapy in the cohort of Chinese patients. In this MEMS-based study, all patients had a history of coronary heart disease (CHD) events or CHD-equivalent risk (Cheng et al., 2004). One reason can be that patients do not perceive the supplementation therapy with Ca/D as much important compared to statin therapy in secondary prevention. The supplementation can be viewed as if it “only” accompanied specific antiresorptive treatment (ibandronate) which is believed to be the main medication that reduces fracture risk. In the study of Cheng et al. adherence slightly decreased during the first months and tended to normalize from the third month onwards (Cheng et al., 2004). This initial decrease in adherence could be explained by the inclusion criteria i.e., patients who had been initiated on statin monotherapy for <12 months. Their cohort was therefore probably closer to the beginning of treatment than our treatment-experienced group and the initial decline in adherence behavior toward statins can be more apparent.

In the MEMS-based study of adherence (Parker et al., 2007) including 145 participants within 2 months of initiating warfarin therapy, adherence was around 80% and changed over time. Authors observed initial worsening over the first 6 months of monitoring, which was followed by improvement beyond 6 months. Similarly, compared to our findings, high level of adherence and initial decline could be explained by higher perceived necessity of treatment and inclusion of treatment-naive patients only. The improvement in adherence to warfarin therapy was explained as a result of loss of anticoagulation and potential benefit from interventions.

Within a psychiatric field, as many as 81% of patients with schizophrenia adherent in the first year were adherent in the second year. Adherence status was relatively stable over time, as most patients (77%) maintained the same adherence status in the second year compared to the first year. However, in this large study, a stable adherence was defined in a less stringent way than in our study, therefore the stability of the adherence in our cohort was lower.

The assessment of adherence over time is particularly studied in HIV-positive patients. However, these patients usually come from different socioeconomic background and probably perceive their condition as serious and life-threatening. Not surprisingly, adherence mostly declines with time. A review including studies with predominantly treatment-experienced cohorts cared for in the United States found out that the decline is not consistent across studies (Wilson et al., 2013). The study with HIV-infected adults (Shuter et al., 2012) evaluated adherence to antiretroviral therapy with the use of MEMS in two observational periods in the interval of 3 years; the mean adherence rates were 74 and 69%. In another study, adherence measured by using self-reported interviews was 81% in patients, who were adherent at baseline, compared to 58% in those non-adherent at baseline (Tesoriero et al., 2003). Even though these findings seem similar to ours, comparisons are not appropriate since adherence in HIV-infected patients is a specific problem different from chronic civilizational conditions (such as osteoporosis) mostly perceived as non-life threatening.

The patterns of non-adherence within a 3-month period were very similar after 1 year. During the follow-up the percentage of patients with drug holidays was slightly higher in comparison with the baseline at the expense of single day omission, but the differences were not significant. Over 70% of patients in our study took drug holidays in both rounds. The finding the patients who had used the pill box at baseline also used it at follow-up is in accordance with the basically unchanging “adherence patterns.” The pattern is probably more important and helpful for the patients than physician's instruction associated with the study.

### Limitations and strengths

The main limitation of our study is a relatively small sample size. Pilot and qualitative studies generate signals and hypotheses; no generalization is possible. We wanted to approach the clinical practice as close as possible—to maximally minimize bias regardless a lower number of participants.

The adherence rate observed might be biased by the fact that the patients who gave consent to participate in the study might have been more motivated to adhere to therapy than those patients who refused to participate. The fact that the patients were recruited directly from the Osteocenter could positively affect the final behavior of the sample willing to participate but it is a general problem of such studies.

The strength of our study is that we describe the loss of patients at both baseline and follow-up in detail, and reasons for exclusion, which is important since it shows adherence-related behavior as a whole. Some studies either do not mention the loss of follow-up or simply note that those lost to follow-up were excluded from analysis (Wilson et al., 2013). We observed adherence in treatment-experienced patients and involved only persistent patients, who are compliant with medical check-ups and agreed with their inclusion in the study—they signed the informed consent. With regard to these strict criteria only less than one half of the patients addressed were involved in the study. It is about more than 20% less than e.g., in the study with patients treated with statins (persistent and non-persistent) measuring adherence by means of MEMS (Cheng et al., 2004). In statin study, which comprised 70% of patients the crucial reasons for excluding patients were similar—11 patients refused to participate and 15 transferred drugs to their pill-boxes (Cheng et al., 2004). Contrary to our study, Cheng et al. had also minor problems such as patients referred to clinics other than the study clinics, insistence on using pill-box, loss of the MEMS bottle, and closing the bottle cap properly.

Patients who know they are monitored improve their adherence; this bias is usually referred as Hawthorne effect (Parker et al., 2007; Zeller et al., 2008b; Maqutu et al., 2011; McCambridge et al., 2014). That is why we informed patients in a similar way as in the study of Cheng (Cheng et al., 2004)—a patient agreed with her involvement in the study by means of informed consent. A patient was informed about the purpose of the study, but it was not specified, that the pill bottle is electronic and intended to record adherence (if she did not ask actively). Thus, patients probably did not have suspicion of being monitored, because no patient asked about the purpose of using the unusual bottle. Therefore, we suppose “real-world-non-adherence patterns” were kept to a great extent.

## Conclusion

In conclusion, the adherence was insufficient (around 70%) at baseline and at follow-up and did not change significantly in individual months. The patterns of non-adherence were similar after 1 year, and over two thirds of patients in our study took drug holidays in both rounds. It seems that signing the informed consent acts as bias more than a medical check-up does, but it is necessary to further investigate this phenomenon.

For clinical practice, the following can be summarized: In MEMS-based studies, it seems appropriate to analyse adherence without the initial data, which are probably influenced by bias of signing the informed consent. Diligent and repeated education of patients with osteoporosis regarding an appropriate diet is needed. The importance of sufficient intake of calcium and vitamin D should be emphasized during each medical check-up.

## Author contributions

TT Conceptualized and organized the study. Handled the patient data and prepared the data for analysis. Carried out analysis of the data and statistics. Drafted the manuscript. Prepared the figures. Contributed to interpretation of the results and revised the draft. MV Conceptualized and organized the study. Handled the patient data and prepared the data for analysis. Carried out analysis of the data and statistics. Drafted the manuscript. Contributed to interpretation of the results and revised the draft. VP Conceptualized and organized the study. Conducted patient recruitment. Contributed to interpretation of the results and revised the draft. TH Conceptualized and organized the study. Drafted the manuscript. Contributed to interpretation of the results and revised the draft. YC Prepared the figures. Contributed to interpretation of the results and revised the draft. LF Contributed to interpretation of the results and revised the draft.

## Funding

The study was supported by Charles University in Prague, the projects SVV 260 187 and SVV 260 295, by MH CZ –DRO (UHHK, 00179906) and by the programme PRVOUK P37/11.

### Conflict of interest statement

The authors declare that the research was conducted in the absence of any commercial or financial relationships that could be construed as a potential conflict of interest.
